# mHAT app for automated malaria rapid test result analysis and aggregation: a pilot study

**DOI:** 10.1186/s12936-021-03772-5

**Published:** 2021-05-26

**Authors:** Carson Moore, Thomas Scherr, Japhet Matoba, Caison Sing’anga, Mukuma Lubinda, Phil Thuma, David Wright

**Affiliations:** 1 Department of Chemistry, Vanderbilt University, 1234 Stevenson Center Lane, Nashville, TN 37212 USA; 2 Macha Research Trust, Choma District, Zambia

**Keywords:** Mobile health, Malaria, Rapid diagnostic test, Application development

## Abstract

**Background:**

There are a variety of approaches being used for malaria surveillance. While active and reactive case detection have been successful in localized areas of low transmission, concerns over scalability and sustainability keep the approaches from being widely accepted. Mobile health interventions are poised to address these shortcomings by automating and standardizing portions of the surveillance process. In this study, common challenges associated with current data aggregation methods have been quantified, and a web-based mobile phone application is presented to reduce the burden of reporting rapid diagnostic test (RDT) results in low-resource settings.

**Methods:**

De-identified completed RDTs were collected at 14 rural health clinics as part of a malaria epidemiology study at Macha Research Trust, Macha, Zambia. Tests were imaged using the mHAT web application. Signal intensity was measured and a binary result was provided. App performance was validated by: (1) comparative limits of detection, investigated against currently used laboratory lateral flow assay readers; and, (2) receiver operating characteristic analysis comparing the application against visual inspection of RDTs by an expert. Secondary investigations included analysis of time-to-aggregation and data consistency within the existing surveillance structures established by Macha Research Trust.

**Results:**

When compared to visual analysis, the mHAT app performed with 91.9% sensitivity (CI 78.7, 97.2) and specificity was 91.4% (CI 77.6, 97.0) regardless of device operating system. Additionally, an analysis of surveillance data from January 2017 through mid-February 2019 showed that while the majority of the data packets from satellite clinics contained correct data, 36% of data points required correction by verification teams. Between November 2018 and mid-February 2019, it was also found that 44.8% of data was received after the expected submission date, although most (65.1%) reports were received within 2 days.

**Conclusions:**

Overall, the mHAT mobile app was observed to be sensitive and specific when compared to both currently available benchtop lateral flow readers and visual inspection. The additional benefit of automating and standardizing LFA data collection and aggregation poses a vital improvement for low-resource health facilities and could increase the accuracy and speed of data reporting in surveillance campaigns.

**Supplementary Information:**

The online version contains supplementary material available at 10.1186/s12936-021-03772-5.

## Background

In 2018, the World Health Organization (WHO) reported 228 million cases of malaria and 405,000 deaths [1]**.** Although malaria is endemic in 81 countries, 94% of malaria-attributable deaths occur in Africa. Approximately half of the world’s population is at risk of infection; current literature proposes that these estimates may be significantly underestimating the true burden of disease [1–3]. The WHO relies on national and regional programmes within each country of interest to collect and report epidemiologic data and has published guidelines for operation of these systems [4]**.** From these guidelines, ‘good-quality’ malaria surveillance data are understood to be a dataset that contains diagnostic results for every potential malaria patient, obtained by validated microscopy or rapid diagnostic testing. Additionally, collection of good-quality data ensures that all diagnostic results are classified correctly, are reported in a complete and consistent manner, and that there is a mechanism in place to verify or audit the collected data [4]. These recommendations outline the gold standard for malaria surveillance and data collection.

Within these guidelines however, the distinct objectives of disease control and elimination campaigns necessitate disparate approaches for surveillance. Countries whose disease burden is low are less likely to carry out universal coverage and testing that defines some malaria control campaigns [5]. In general, as disease prevalence in these countries decreases, due to the sweeping interventions and wide-scale testing that accompany control campaigns, these campaigns become more expensive and unwieldy for detection of single cases or very small pockets of disease. Conversely, elimination programmes tend to be characterized by community-based testing at the individual health post level [5, 6]. One popular strategy for this type of programme is active case detection (ACD) [7, 8]. ACD focuses on the detection of individual malaria cases at the community and household level within high-risk populations [6, 9]. In Zambia and other low- and middle-income countries (LMICs) approaching elimination, ACD is manifested in the form of a ‘test and treat’ strategy: febrile patients or at-risk asymptomatic patients are tested for infection and treated accordingly [6, 10, 11]. Additional active detection programmes, such as the reactive screen and treat programme utilized in Southern Province, Zambia, rely on teams of healthcare workers to travel to the household of an index case and screen all individuals within a certain radius [12]. While these techniques have proven effective, active and reactive detection methods have the potential to be expensive and time-consuming, based on the prevalence of disease and the intended length of the programme [13, 14]. As public health stakeholders push for worldwide elimination, many LMICs are interested in simple, effective and inexpensive methods for improving collection and aggregation of surveillance data collected by these programmes.

With the current tools, even the most well-equipped health outpost in a high-burden, low-resource setting may struggle to meet WHO goals for surveillance data. This is particularly true when attempting to implement a possibly infrastructure-heavy intervention, such as reactive screen and treat. Healthcare in LMICs varies widely, as many nations have different healthcare provisions; care often consists of treatment from community health workers (primary care) [15–17]. In certain low-income nations or regions, healthcare infrastructure is further strained by uneven access to more involved interventions [17]. To this point, Africa has the lowest average health worker density in the world, with 90% of countries reporting below 10 medical doctors per 10,000 people [18]. While factors such as travel and cost can be prohibitive, difficulty in accessing potentially malaria-positive patients with diagnostic tools is also compounded by the lack of available healthcare workers [19, 20].

In the last decade, mobile health (mHealth) has become a popular tool to address the challenges associated with traditional healthcare systems. mHealth is the utilization of mobile communication technology to connect users directly with healthcare services and providers. The effect has been especially powerful in areas underserved by traditional brick-and-mortar medical facilities, such as LMICs [21, 22]. This is well-illustrated by a 2013 HIV monitoring programme in Mozambique [23]. The Mozambique Ministry of Health implemented web-based data collection for decentralized HIV monitoring in which point-of-care CD4 count devices equipped with wireless capabilities were distributed to health facilities across the country. Using this network, healthcare workers were able to relay surveillance data directly to the Ministry of Health, including number of tests performed each day, any errors encountered, and quality control checks. As the programme scaled up, testing errors at the point of care (including failed controls and testing reagent loss) were reduced from 13 to 5% and have remained stable, increasing confidence in the reliability and quality of the decentralized testing facilities across the country.

**Fig. 1:**
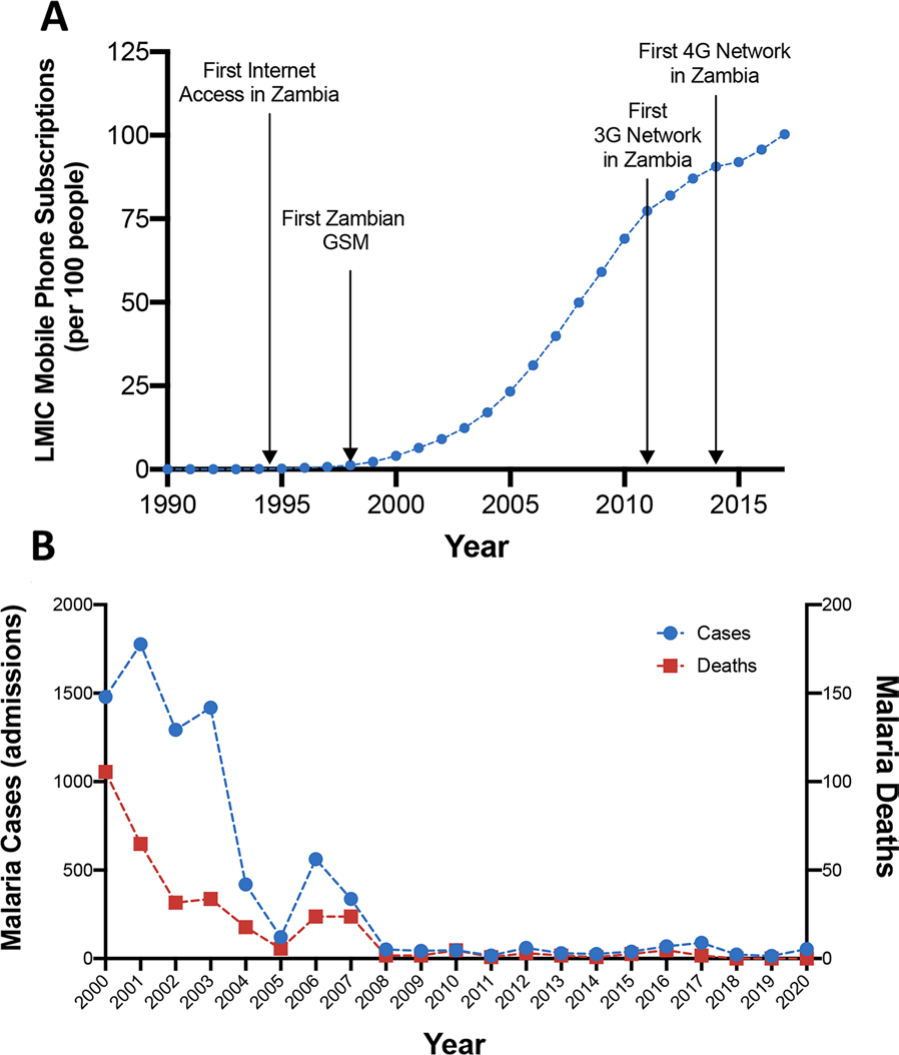
**A** The number of mobile phone subscriptions per 100 people in World Bank low and middle-income countries from 1990 to 2017. Arrows represent important developments in the telecommunications landscape of Zambia. **B** Malaria admissions and deaths at the Macha Hospital Children’s Ward after the initiation of a reactive screen and treat campaign in 2002–2003

An increasing number of mHealth interventions have followed as a natural consequence of the high penetration of mobile phones into LMICs; subsequent developments in mobile network capacity have accelerated over the last two decades (Fig. 1A)[24]. Studies have already been undertaken to investigate the role of mobile phones in malaria case management [25–27]. Many of these interventions utilize short message service (SMS) to convey reminders regarding drug adherence or report collected data on rapid diagnostic test (RDT) detection rates. However, there has been little application of mobile phones in analysis and automated data recording and reporting. In an analysis of several mobile malaria RDT analysis applications, data from over 1,600 RDTs were collected and recorded by hand on paper data collection sheets before being imported manually to Microsoft Excel [28]. While these applications were judged to have performed as well as the human eye in detecting positive and negative RDTs, they lacked the ability to automatically collate critical healthcare data. The challenges involved with developing confidential, accurate and integrated systems for healthcare information may discourage developers from attempting to go beyond surface-level mHealth interventions, and some currently available applications are hampered by poor sensitivity compared to visual interpretation of tests [28, 29].

Mobile technology is uniquely poised to improve malaria surveillance campaigns. In this paper, the mobile health and treat system (mHAT) is presented as one solution to the challenges that elimination campaigns currently face. mHAT is a mobile phone-based web application that utilizes image processing software to automatically detect test lines on commercially available RDTs and provide semi-quantitative results. These results are standardized within the platform and stored in a secure research database (REDCap) for automated downstream analytical surveillance efforts. For this pilot study, data collected using the mHAT app are compared to current data collection and surveillance practices in a highly functioning LMIC healthcare setting in Southern Province, Zambia, which has had renowned success with reactive screen and treat campaigns (Fig. 1B). The mHAT system takes advantage of available healthcare infrastructure through the use of RDTs and community healthcare workers, while also leveraging the ubiquity of mobile phones and their native reporting capabilities for surveillance with improved accuracy and speed.

## Methods

### mHAT application

The mHAT app is a mobile-friendly, web application hosted on a development server at Vanderbilt University, Nashville, USA. For the duration of the study it was accessible in any web browser by its public IP address.

mHAT was designed as a web application (hosted by a webserver and running in the browser of a mobile device) rather than a native application that is downloaded and runs locally on the device. This decision was guided by the expected use case, and was influenced by the broad device compatibility, and the ease and speed with which software updates can be deployed, of web applications. The architecture described above can work on any web-connected device (i.e., phones, tables, laptops, desktops) and interchangeably across operating systems. The mHAT app does require network connectivity, but new developments that enable progressive web apps will allow future versions of the software to have similar functionality offline as well.

Computer vision for analysis of RDTs is a substantial endeavour. These algorithms must be tolerant of lighting and background variations and different camera resolutions. A more thorough description of the technical implementation of a computer vision algorithm is detailed in Additional file 1. Here, the app use is briefly described. First, users input any information they would like to associate with this test (e.g., case ID, test ID, any other notes). From within the app, the user presses a button to take a photograph of the RDT they would like to analyse. Prior to taking the photograph, the web app asks to access the user’s GPS coordinates to assist with spatial infectious disease surveillance. Preliminary image processing is performed and determines if the photograph must be retaken, or if it is suitable for full analysis. If the preliminary photograph is suitable, a preview image is presented to the user with annotations of key test features. The user has the choice to accept this image and proceed with analysis, or they can elect to take another photograph and restart the process. If the user proceeds with analysis, the full results are presented to the user, including: a qualitative ‘Positive’/ ‘Negative’ result as well as the numerical values for test line signal, control signal and signal ratio. The result is transferred to a custom REDCap (a widely used, customizable research electronic database) [30–32] project for storage, along with the user that uploaded the test information, GPS coordinates, and a timestamp. This information facilitates spatio-temporal surveillance, as well as allowing national healthcare systems to observe the locations of individual community healthcare workers and their teams. A detailed outline of the development of the mHAT software and image processing analysis has been included in the Additional file 1: Fig. S1).

For any new test, computer vision and image processing features must be experimentally optimized. This is a result of the different shapes of cassettes, the colours of the test and control line signals, markings and brandings on the test cassettes, different spatial differences during manufacturing of each test. However, this process is relatively straightforward, and with minimal effort can enable the use of the software to analyse a variety of tests. This optimization was performed for several malaria tests (including multiplexed tests with multiple test lines), multiple HIV test kits, schistosomiasis test kits, pregnancy and ovulation test kits. Since the algorithm utilizes feature recognition, it has the potential to automatically detect the type of test from the photograph, without additional user input. This would have obvious utility for organizations that heavily rely on point-of-care testing.

### Laboratory RDT image-processing, training and validation

Initial image-processing optimization was performed using SD Bioline Malaria Ag P.f. tests (Standard Diagnostics, South Korea). D6 *Plasmodium falciparum* parasite was cultured in-house. Human whole blood (pooled, citrate phosphate dextrose anticoagulated) was purchased from BioIVT. Samples were prepared by spiking whole blood with D6 parasite culture at 5000 p/μL and diluting serially by a factor of 2.5. The parasite-positive dilution series consisted of 6 total concentrations: 5,000, 2,000, 800, 320, 128, and 51.2 parasites per μL of whole blood. A whole blood sample with no parasite culture was used as a negative control. These mock specimens were applied to the RDTs, which were run according to manufacturer’s instructions. Briefly, a 5-μL sample was added to the specimen well of the test; 4 drops of proprietary running buffer were then added to the diluent well, and the test was allowed to develop for 15 min at room temperature. After 15 min, photos were taken in the web-based mHAT app on both an iPhone 8 + (Apple, USA) and Samsung Galaxy J3 (Samsung, South Korea). All tests were analysed in method triplicate, resulting in 3 unique RDTs that were imaged for each parasite density. All mHAT results were automatically recorded and stored in a pre-configured REDCap project. Immediately after mobile phone imaging, each test was removed from its plastic casing and the nitrocellulose lateral flow test strips were analysed on a Qiagen ESEQuant lateral flow reader (QIAGEN Lake Constance GmbH, Stokach, Germany). Lateral flow readers are useful laboratory- or clinic-based tools for objective RDT analysis [33]. However, these instruments require continuous power, trained personnel, and cost thousands of dollars, making them unsuitable for field usage in remote settings. There are other optical approaches for RDT analysis [34, 35], but these have been demonstrated only in research settings and are not commercially available. Therefore, the QIAGEN reader represents one such tool that has been used previously as a benchmark for lateral flow assay analysis [36] and is commercially available.

### RDT collection and imaging in Southern Zambia

**Fig. 2:**
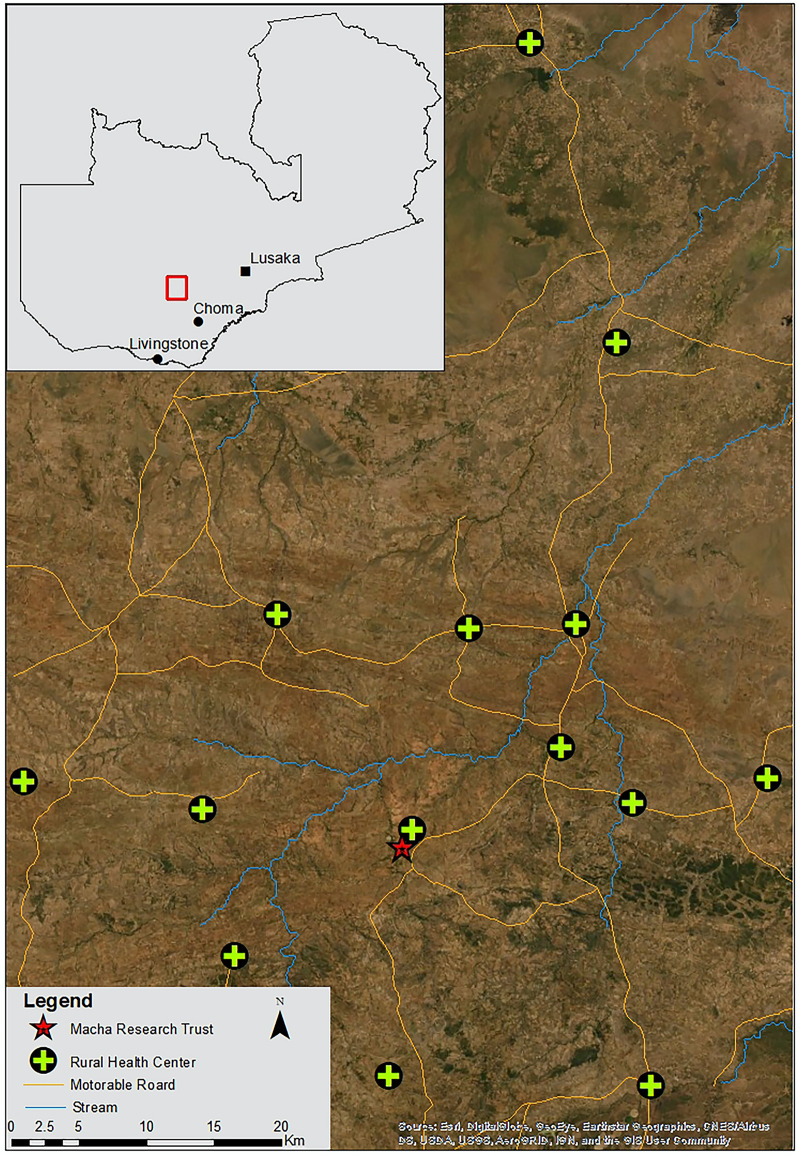
Map showing Macha Research Trust (MRT, red star) and each of the 14 rural clinics within the MRT catchment area (green crosses)

Macha Research Trust (MRT) relies on several teams of researchers, physicians and community healthcare workers to collect surveillance data and provide treatment for the population in and surrounding Macha, using a hub-and-spoke model of satellite clinics. Aggregate surveillance data are validated quarterly by an independent team of MRT researchers, and collected weekly from each of the 14 MRT outposts in the area (Fig. 2).

RDT collection was performed in collaboration with the outpatient clinic (OPC) triage team and the field research team at MRT (Macha, Zambia), the latter as part of an existing Institutional Review Board-approved malaria epidemiology study. All patients were first screened by underarm temperature. In the OPC, SD Bioline Malaria Ag P.f. tests were performed for all patients presenting with a temperature at or above 37 °C. In the field, SD Bioline Malaria Ag P.f RDTs were performed for all patients presenting with a fever at or above 38 °C.

Upon completion of an RDT, the administering MRT clinical worker recorded the result in a physical ledger along with patient name age, village, history of travel, weight, blood pressure, pulse, and temperature. The data were not available to the mHAT app team. As the MRT clinicians recorded the necessary data for their reports, the RDT was imaged using the mHAT app and the result was recorded and stored in the REDCap database. While additional time elapsed may change the amount of blood clearance on RDTs, resulting in changes in contrast between the test line and the background of the nitrocellulose, RDTs were analysed by visual inspection immediately prior to being analysed by mHAT. The time from visual inspection of the RDT to successful mHAT analysis was under 1 min in most cases, with the exception of early tests using the first iteration of the mHAT app. This elapsed time was determined to be unlikely to introduce any significant error. The number of image re-takes prior to analysis and the binary mHAT result were additionally recorded. The image orientation and photo acceptance features were improved iteratively over the course of this pilot study.

### mHAT optimization for field performance

A controlled laboratory environment is an idealized setting for a mobile application that uses computer vision to analyse global health RDTs. To mitigate potential errors in photography resulting from an uncontrolled field setting, the study incorporated: (1) an automated checkpoint where the algorithm attempts to automatically determine if a photograph is satisfactory for processing; and, (2) a manual checkpoint where a user can determine if the photograph should be retaken. In addition, it was anticipated that adjustments to the image-processing algorithms may improve field performance (Additional file 1: Fig. S2).

### Analysis of current data collection and aggregation systems

As one of the leading medical research sites in Southern Zambia, and an active participant in the Zambian malaria elimination effort, MRT has a well-developed system for collection and verification of malaria RDT results. Data collection is performed using a network of clinic-based healthcare workers who summarize recorded data from clinic record legers at the end of each week. Every Monday, one healthcare provider from each clinic aggregates the data from the past 7 days and enters it into an SMS message. This SMS is then sent to a member of the MRT data aggregation team who collates the data from the satellite sites into a single spreadsheet, which is then manually entered into a REDCap project.

Verification of this SMS data is performed quarterly. To verify the data, the verification team travels to each of the satellite clinics and compares reported data to the physical clinic ledger. Every physical entry describing either positive and negative RDT result is counted and the verification team notes the date and total for each week. All changes to the data are recorded on a physical copy of the SMS aggregate spreadsheet, and changes are recorded digitally upon return to the laboratory. If the tallies are divergent, the verification team resolves the disparity through a record re-count at the satellite clinic. The final corrected tally is noted in red ink on a physical copy of the collated SMS data. The verified and corrected data are then uploaded from Microsoft Excel to REDCap.

Observational analysis of this system was performed by collecting and evaluating the corrected spreadsheets from January 2017-February 2019. The number of corrections was determined by comparing the number of correct (unaltered) data points to the number of corrected (altered) data points for each clinic over time. Date and time data were collected from SMS records between November 2018 and February 2019.

### Statistical analysis

Experiments to determine the limit of detection were performed in triplicate, with the average and standard deviation being calculated at each parasite density. The limit of detection was calculated using 3SD_blank_ divided by the slope of the regression of the linear region of the data.

## Results

### mHAT RDT reader performance validation

**Fig. 3:**
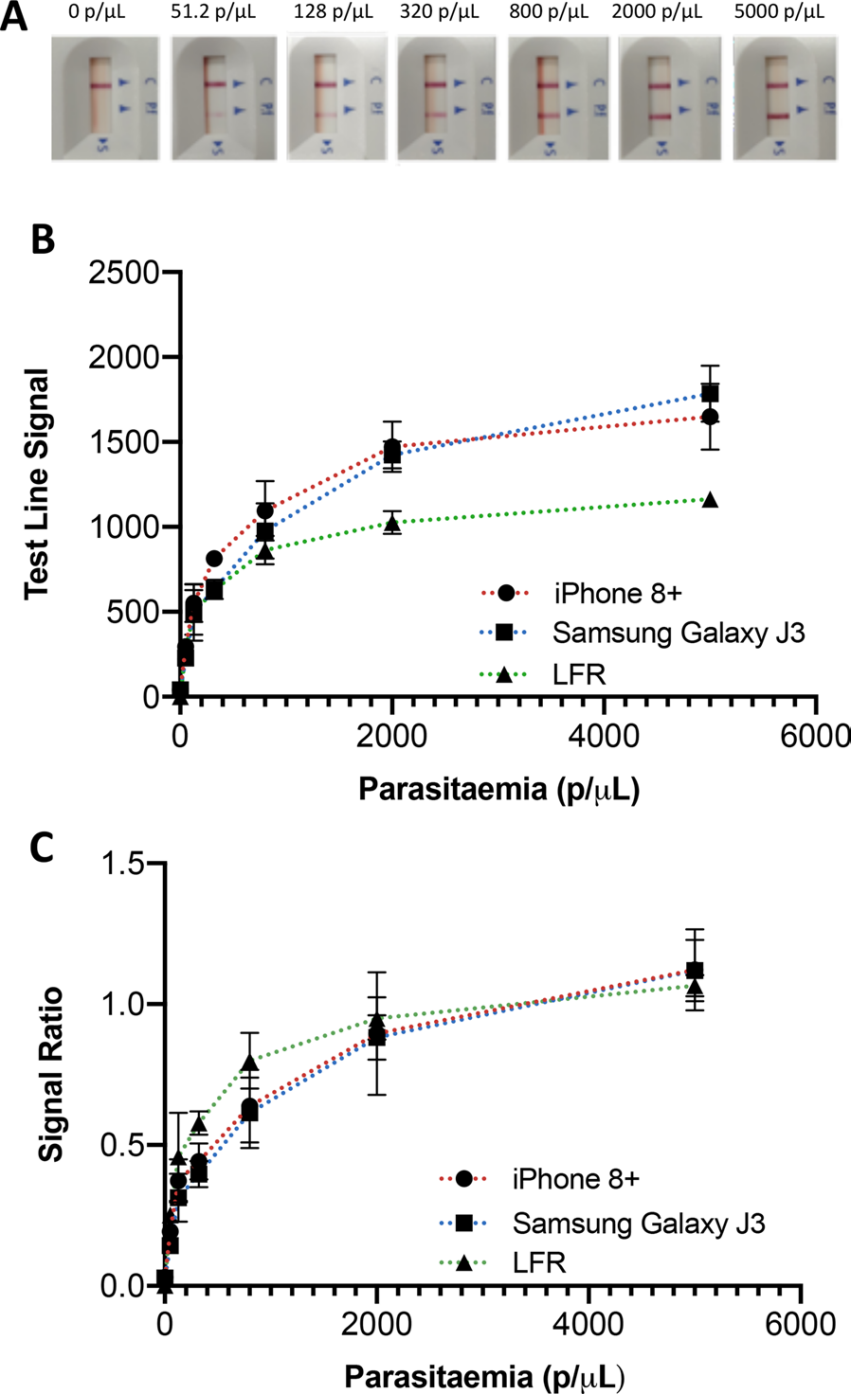
iPhone images of the RDT titration series used to determine the mHAT limit of detection (**A**). Limit of detection analyses were performed in parasitized blood using both test line signal alone (**B**) and test line to control line signal ratio (**C**) on two mobile phones and a commercial lateral flow reader. Analytical sensitivity for the mHAT app on iOS and Android platforms is on the same order as the sensitivity of the commercial lateral flow reader

The limit of detection for the mHAT app was determined to be comparable to a commercial lateral flow reader (Fig. 3A). For all devices used, signal intensity monotonically increases as the parasite density in the sample increases; at higher concentrations the rate of increase slows and the test line signal intensity begins to plateau. Both test line signal and the ratio of test line and control line signal were considered as threshold metrics. Using the test line signal, the limits of detection were found to be 15.0 ± 3 parasites/μL, 14.9 ± 4 parasites/μL and 6.12 ± 2 parasites/μL using the iPhone 8 + , Samsung Galaxy J3 and ESEQuant LFR, respectively (Fig. 3B). When considering signal ratio (test line signal over control line signal) as the read-out measure, the mHAT app limits of detection increased slightly but not significantly (Fig. 3C). The limits of detection were found to be 23.2 ± 8 parasites/μL and 20.9 ± 6 parasites/μL while the EQEQuant LFR was found to have a limit of detection of 6.15 ± 2 parasites/μL.

### Quantification of data reporting and aggregation in active case detection

**Fig. 4:**
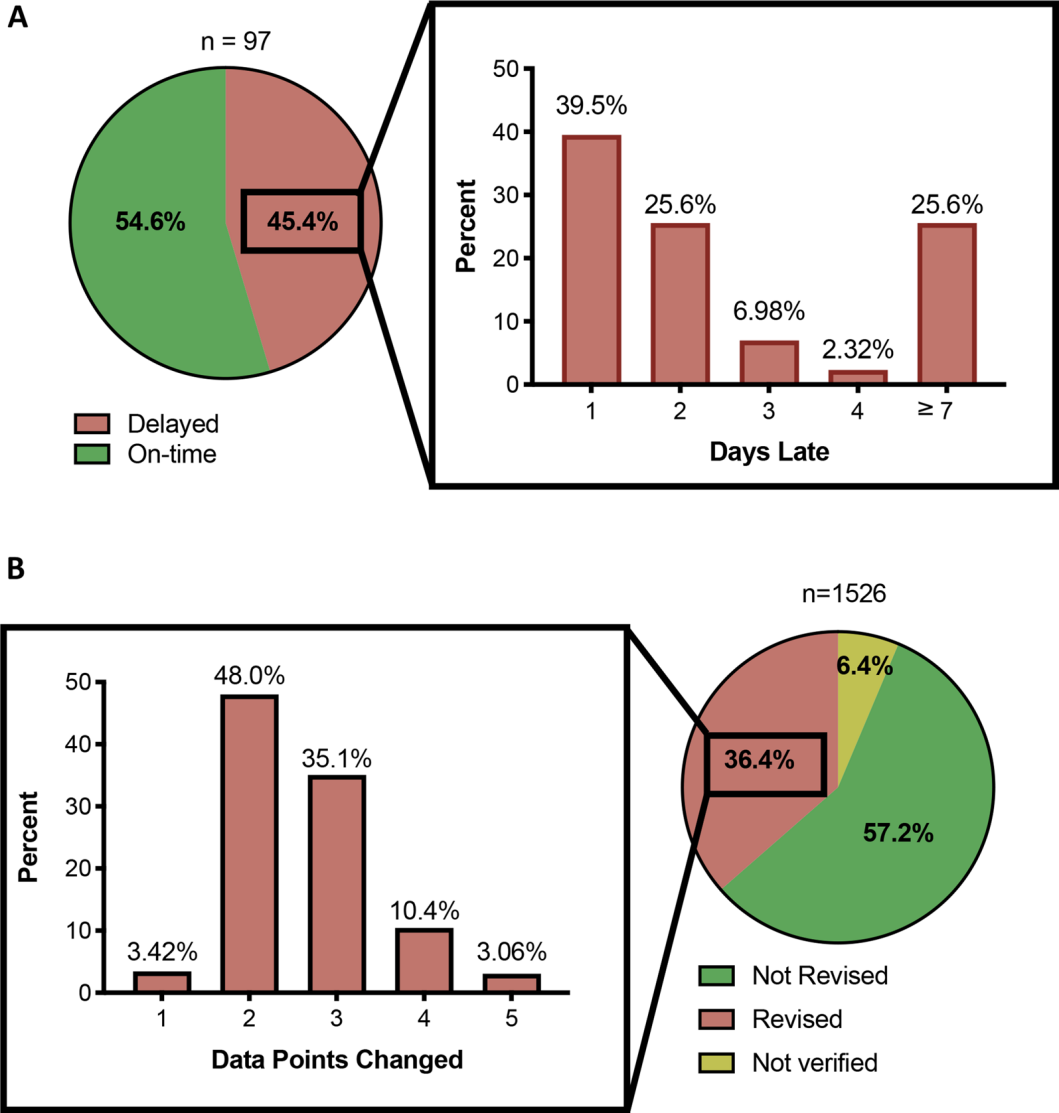
**A** Percentage of reported surveillance data that was received after the expected date of arrival. The box to the right shows the histogram of late reports binned by the number of days past the due date at which the data was received. **B** Percentage of recorded surveillance data that was revised upon review by the MRT remote verification team. The box to the left shows the histogram of revised data points binned by the number of data points altered per report

For the 14 satellite clinics operated by MRT, surveillance data are aggregated by mobile messaging. MRT is not immune to the variety of challenges that arise with data collection in low-resource settings, and this work sought to quantify the impact of these obstacles. Physical records of malaria RDT data are summarized by community healthcare workers (CHWs) at each clinic and reported by SMS every Monday. To assess the temporal efficacy of this system, SMS records were collected spanning the period between November 2018 and February 2019. Of the 96 weeks of data analysed, only 54.6% of the data packages were received on the Monday expected (Fig. 4A). When data were received late, typically within 1–2 days of when expected, but over 25% of the time the report was received over a week late.

When analysing physical data from January 2017 to February 2019, it was found that most weekly data packets were reported correctly and required no revision by the verification team; however, 36% of the weekly data points did require correction (Fig. 4B). Of the weeks in which data were corrected, most required at least two data points to be corrected, and almost half required three or more points to be corrected. The most common error observed was incorrect entry, where the counts were correct but correct numbers for two categories (e.g. positive RDT, negative RDT) had been exchanged. Approximately 6% of data were not verified at the time of analysis due to external causes, including environmental factors that limited the ability of the verification team to reach clinics, physical damage to clinic records, or lost records.

### mHAT application field performance

The most common method for RDT analysis in the field is visual inspection. It has been demonstrated that visual inspection of RDTs varies with CHW experience [37]. The healthcare workers in and around MRT have a high level of familiarity with malaria RDTs, and thus, for this study the mHAT app was compared to visual interpretation of RDTs by these experienced CHWs as a gold standard. All RDTs were identified as positive or negative by a CHW at the point of care, and validated by the researcher using the mHAT app. Iterative improvement of the image analysis software was required over the span of the trial to account for differences between photo quality in a controlled laboratory setting and the field (Additional file 1: Fig. S2).

**Fig. 5:**
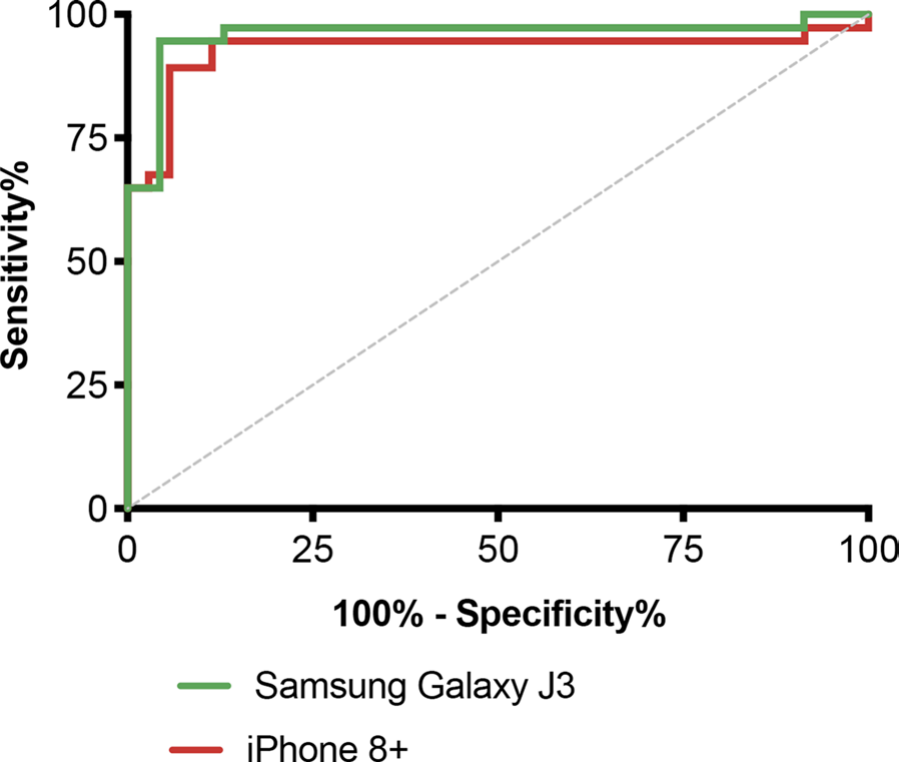
Receiver operator characteristic (ROC) curves for the mHAT app when compared to visual interpretation of rapid diagnostic tests by an experienced healthcare worker

In this analysis, both the signal at the test line and the ratio of test line and control line signal were evaluated as potential read-out values and ideal thresholds for each signal metric were determined (Fig. 5, Additional file 1: Fig. S3). Both values were assessed on iOS and Android devices.

Test line to control line signal ratio was utilized as the determinant for mHAT test results. While significant batch-to-batch variation between RDTs was not observed, the decision to use signal ratio as a metric rather than test line signal alone offers protection from this issue, as has been documented previously [38]. Sensitivity for signal ratio analysis on iOS devices was found to be 94.6% (CI 81.8, 99.3) and specificity was 88.6% (CI 73.3, 96.8). Using an Android device, sensitivity was 94.6% (CI 81.8, 99.3) and specificity was 95.6% (CI 78.1, 99.9). When both device types were analysed, the combined sensitivity was then observed to be 94.6% (CI 81.1, 99.3) and specificity was 88.6% (CI 73.3, 96.8). Positive predictive value (PPV) and negative predictive value (NPV) for the mHAT app were observed to be 0.923 and 0.952, respectively.

## Discussion

In this study, mHAT, a mobile health tool for improving surveillance campaigns through a mobile-friendly web application that automates the analysis and reporting of point-of-care diagnostic tests, was developed and analysed. This pilot study was performed in Macha, Zambia in order to understand how the app could complement existing surveillance campaigns. The MRT has had success in decreasing the malaria burden in the region through active and reactive case detection and has implemented the standards set out by WHO with regard to data collection, reporting and verification [10]. To ensure reporting data accuracy, MRT has established an independent data validation team, which works to authenticate collected surveillance data on a quarterly basis. It was anecdotally known that data collection before validation was occasionally subject to time delays and misinformation, possibly caused by input from multiple disparate healthcare workers or physical damage or loss of records, but this delay had not been previously quantified. While the validation technique used by MRT has proven to be successful, many low-resource settings will not have access to resources of this type and may not be able to validate data with the required level of rigour, leaving their reporting open to delays and inaccuracies. These delays and inaccuracies could be due to any number of factors, including the number of patients being seen at each clinic, seasonal environmental factors, or weak mobile phone signal for SMS data aggregation.

The mHAT app was utilized, in both a laboratory setting and in a small pilot field study, to perform automated objective RDT analysis, data collection and reporting. mHAT was found to have a limit of detection as low as 15 parasites/μL of whole blood, comparable to that of a commercial lateral flow reader in the laboratory. The mHAT app was also shown to have little variation between mobile phone types, with comparable limits of detection observed not only between mobile phones and the commercial lateral flow reader, but also between the two distinct brands. The two mobile devices used in this study have substantially different camera hardware (iPhone 8 + , 12 MP camera with dual lens; Samsung Galaxy J3, 8 MP camera), and previous work has shown that similar algorithms are also effective when using earlier model devices that have lower resolution cameras [37]. Additionally, mHAT performed with high sensitivity (> 90%) and specificity (> 85%) compared to visual inspection of tests in the field. However, it is necessary to note that malaria RDTs are generally intended to be qualitative tests, reliant on the interpretation of the presence or absence of coloured signals at the test line [37, 39, 40]. Although test line signal intensity generally tracks with parasite density, the result should be considered only a semi-quantitative analysis of the RDT itself. Despite this, the app is ideal for low-resource uses because it requires minimal training, provides results in seconds [41], and automatically collates RDT data into a single accessible database without the errors that can arise in manual data recording. While REDCap was utilized for data storage, this platform could easily be modified for compatibility with any existing record system, including DHIS2, OpenMRS or mUZIMA.

In addition to the flexibility mHAT offers, the app has several strengths, including minimal up-front investments in trained personnel or infrastructure and relatively low operating costs. The app requires no further instrumentation (e.g., readers, dongles, phone attachments) than a camera-enabled smartphone, which, as outlined earlier, can easily be found in many LIMC settings (Fig. 1). Data use for this app is low, and can cost below US$1 per week [41]. However, the main limitation of the current version of the mHAT software is that it does require a consistent internet connection, which may not be available in all settings where RDT analysis is performed. In order to address this shortcoming, both mobile and web-based applications will be developed that are capable of offline operations and asynchronous data transfer for use in instances where dependable internet access cannot be maintained.

Although the focus in this publication centered on the accuracy of the results generated by the mHAT app, and the comparison to traditional surveillance reporting systems, there are many additional features that make mHAT a complete surveillance tool. These features include: healthcare worker training guides, case maps, timelines, calendars, a user hierarchy which allows for delineation of privileges, administrator access and an administrator dashboard for user monitoring, secure authentication, transmission, and storage of data. Combined, these features represent a rapid, fully functional, user-friendly mobile healthcare experience. The targeted use-case for this application was for low-resource disease surveillance. However, with little additional effort, it could have immediate relevance to the current COVID-19 pandemic, which has resulted in dozens of RDTs receiving Emergency Authorization Use status from the US Food and Drug Administration. This further underscores the importance of support for global health initiatives and their ability to drive innovation that can have unexpected positive impacts, even in domestic settings.

## Conclusion

Diagnostic data collection and aggregation is a major challenge in malaria surveillance efforts. Mobile health initiatives are one approach to improving the timeliness and accuracy of field data reporting. In this study, the mHAT app was developed and field-tested to objectively interpret malaria point-of-care diagnostic tests. The mHAT app was found to compare favourably to both the gold standard for field and laboratory-based RDT analysis. The numerous benefits of standardized LFA analysis and automated data aggregation represent a critical improvement for low-resource health facilities and could drastically improve the accuracy and speed of broad, long-term surveillance campaigns for malaria and other diseases.

## Supplementary Information

**Additional file 1: Figure S1.** The image analysis steps used in the mHAT application. **Figure S2.** Number of photo retakes needed to achieve an mHAT accepted image on any device during the first two days of the field study, and the last two days of the field study. The mean number of retakes needed in the first two days was 3.500 (5.46), compared to 1.284 (0.670) on the final two days of the field trial. **Figure S3.** Receiver operator characteristic (ROC) curves for the mHAT application, when compared to visual interpretation of RDTs by an experienced healthcare worker, using test line signal as the reporting metric. For iOS devices, sensitivity using test line signal was found to be 91.9% (CI 78.1–98.3%) and specificity was found to be 91.4% (CI 76.9–98.2%). Using an Android device, sensitivity with test line signal was found to be 97.3% (CI 85.8–99.9%), and specificity 95.6% (CI 78.1–99.9%). The combined sensitivity for iOS and Android devices was found to be 91.9% (CI 78.7–97.2%) and specificity was 91.4% (CI 77.6–97.0%). **Figure S4.** Percent of tests that were observed to have each of the 4 mHAT errors: blood failing to clear from the nitrocellulose membrane (blood clearance), missing control line, physical damage to the test or casing (test defect), or interfering environmental defects.

## Data Availability

The datasets used and analysed during the current study are available from the corresponding author (TS) on reasonable request.

## Abbreviations

mHAT: Mobile health and treatment
RDT: Rapid diagnostic test
WHO: World Health Organization
ACD: Active case detection
mHealth: Mobile health
LMICs: Low- and middle-income countries
MRT: Macha Research Trust
IRB: Institutional Review Board
OPC: Outpatient clinic
CHW: Clinical health worker
PPV: Positive predictive value
NPV: Negative predictive value
LFA: Lateral flow assay
SMS: Short messaging service

## References

[1] WHO. World malaria report (2019). Geneva, World Health. Organization.

[2] Alonso P, Noor AM (2017). The global fight against malaria is at crossroads. Lancet.

[3] Editorial (2011). Malaria: control vs elimination vs eradication. Lancet.

[4] WHO (2018). Malaria surveillance, monitoring & evalution: a reference manual.

[5] Bridges DJ, Winters AM, Hamer DH (2012). Malaria elimination: surveillance and response. Pathog Glob Health.

[6] WHO (2012). Disease surveillance for malaria control: an operational manual.

[7] Wickremasinghe R, Fernando SD, Thillekaratne J, Wijeyaratne PM, Wickremasinghe AR (2014). Importance of active case detection in a malaria elimination programme. Malar J.

[8] Smith Gueye C, Sanders KC, Galappaththy GN, Rundi C, Tobgay T, Sovannaroth S (2013). Active case detection for malaria elimination: a survey among Asia Pacific countries. Malar J.

[9] Sturrock HJW, Novotny JM, Kunene S, Dlamini S, Zulu Z, Cohen JM (2013). Reactive case detection for malaria elimination: real-life experience from an ongoing program in Swaziland. PLoS ONE.

[10] Deutsch-Feldman M, Hamapumbu H, Lubinda J, Musonda M, Katowa B, Searle KM (2018). Efficiency of a malaria reactive test-and-treat program in Southern Zambia: a prospective, observational study. Am J Trop Med Hyg.

[11] Silumbe K, Chiyende E, Finn TP, Desmond M, Puta C, Hamainza B (2015). A qualitative study of perceptions of a mass test and treat campaign in Southern Zambia and potential barriers to effectiveness. Malar J.

[12] Searle KM, Hamapumbu H, Lubinda J, Shields TM, Pinchoff J, Kobayashi T (2016). Evaluation of the operational challenges in implementing reactive screen-and-treat and implications of reactive case detection strategies for malaria elimination in a region of low transmission in southern Zambia. Malar J.

[13] Graz B, Willcox M, Szeless T, Rougemont A (2011). “Test and treat” or presumptive treatment for malaria in high transmission situations? A reflection on the latest WHO guidelines. Malar J.

[14] Zikusooka CM, McIntyre D, Barnes KI (2008). Should countries implementing an artemisinin-based combination malaria treatment policy also introduce rapid diagnostic tests?. Malar J.

[15] WHO (2018). Model list of essential in vitro diagnostics.

[16] WHO (2017). Tracking universal health coverage: global monitoring report.

[17] Okereke M, Ukor NA, Adebisi YA, Ogunkola IO, Iyagbaye EF, Owhor GA (2021). Impact of COVID-19 on access to healthcare in low- and middle-income countries: current evidence and future recommendations. Int J Health Plann Manag.

[18] WHO. World Health Statistics (2019). monitoring health for the sdgs, sustainable development goals.

[19] Bohren MA, Hunter EC, Munthe-Kaas HM, Souza JP, Vogel JP, Gülmezoglu AM (2014). Facilitators and barriers to facility-based delivery in low- and middle-income countries: a qualitative evidence synthesis. Reprod Health.

[20] Varela C, Young S, Mkandawire N, Groen RS, Banza L, Viste A (2019). Transportation barriers to access health care for surgical conditions in Malawi a cross sectional nationwide household survey. BMC Public Health.

[21] Steinhubl SR, Muse ED, Topol EJ (2013). Can mobile health technologies transform health care?. JAMA.

[22] Istepanian RSH, Al-Anzi T. Mobile health (m-health). In: Dagan Feng D, editor. Biomedical Information Technology. 2nd ed. Academic Press; 2020. p. 717–33.

[23] Jani LV, Quevedo JI, Tobaiwa O, Bolinger T, Sitoe N, Chongo P (2016). Use of mobile phone technology to improve the quality of point-of-care testing in a low-resource setting. AIDS.

[24] International Telecommunication Union. Mobile cellular subscriptions (per 100 people). World Telecommunication/ICT Development Report and Database. https://data.worldbank.org/indicator/IT.CEL.SETS.P2.

[25] Porter G (2016). Mobilities in rural Africa: new connections, new challenges. Ann Am Assoc Geographers.

[26] Zurovac D, Talisuna AO, Snow RW (2012). Mobile phone text messaging: tool for malaria control in Africa. PLoS Med.

[27] Prue CS, Shannon KL, Khyang J, Edwards LJ, Ahmed S, Ram M (2013). Mobile phones improve case detection and management of malaria in rural Bangladesh. Malar J.

[28] Visser T, Ramachandra S, Pothin E, Jacobs J, Cunningham H, Ke Menach A (2021). A comparative evaluation of mobile medical APPS (MMAS) for reading and interpreting malaria rapid diagnostic tests. Malar J..

[29] Wallis L, Blessing P, Dalwai M, Shin SD (2017). Integrating mHealth at point of care in low- and middle-income settings: the system perspective. Glob Health Action.

[30] Harris PA (2012). Research Electronic Data Capture (REDCap) - planning, collecting and managing data for clinical and translational research. BMC Bioinformatics.

[31] Harris PA, Taylor R, Thielke R, Payne J, Gonzalez N, Conde JG (2009). Research electronic data capture (REDCap)—a metadata-driven methodology and workflow process for providing translational research informatics support. J Biomed Inform.

[32] Harris PA, Taylor R, Minor BL, Elliott V, Fernandez M, O'Neal L (2019). The REDCap consortium_Building an international community of software platform partners. J Biomed Inform.

[33] Wild D (2013). The immunoassay handbook: theory and applications of ligand binding.

[34] Wang Y, Qin Z, Boulware DR, Pritt BS, Sloan LM, González IJ (2016). Thermal contrast amplification reader yielding 8-fold analytical improvement for disease detection with lateral flow assays. Anal Chem.

[35] DeSousa J, Jorge M, Lindsay H, Haselton F, Wright D, Scherr T (2021). Inductively coupled plasma optical emission spectroscopy as a tool for evaluating lateral flow assays. Anal Methods..

[36] Markwalter CF, Kantor AG, Moore CP, Richardson KA, Wright DW (2019). Inorganic complexes and metal-based nanomaterials for infectious disease diagnostics. Chem Rev.

[37] Scherr TF, Gupta S, Wright DW, Haselton FR (2016). Mobile phone imaging and cloud-based analysis for standardized malaria detection and reporting. Sci Rep.

[38] Colley DG, King CH, Kittur N, Ramzy RMR, Secor WE, Fredericks-James M (2020). Evaluation, validation, and recognition of the point-of-care circulating cathodic antigen, urine-based assay for mapping *Schistosoma mansoni* infections. Am J Trop Med Hyg.

[39] Tangpukdee N, Duangdee C, Wilairatana P, Krudsood S (2009). Malaria diagnosis: a brief review. Korean J Parasitol.

[40] Mukkala AN, Kwan J, Lau R, Harris D, Kain D, Boggild AK (2018). An update on malaria rapid diagnostic tests. Curr Infect Dis Rep.

[41] Scherr TF, Moore CP, Thuma P, Wright DW (2020). Evaluating network readiness for mHealth interventions using the Beacon Mobile Phone App: application development and validation study. JMIR MHealth UHealth.

