# Supplementary material for: mHAT app for automated malaria rapid test result analysis and aggregation: a pilot study
Source: Malar J. 2021 May 26;20:237. doi: 10.1186/s12936-021-03772-5 (PMC8153521; doi:10.1186/s12936-021-03772-5)
Supplement: Supplementary file 1 — Additional file 1: Figure S1. The image analysis steps used in the mHAT application. Figure S2. Number of photo retakes needed to achieve an mHAT accepted image on any device during the first two days of the field study, and the last two days of the field study. The mean number of retakes needed in the first two days was 3.500 (5.46), compared to 1.284 (0.670) on the final two days of the field trial. Figure S3. Receiver operator characteristic (ROC) curves for the mHAT application, when compared to visual interpretation of RDTs by an experienced healthcare worker, using test line signal as the reporting metric. For iOS devices, sensitivity using test line signal was found to be 91.9% (CI 78.1–98.3%) and specificity was found to be 91.4% (CI 76.9–98.2%). Using an Android device, sensitivity with test line signal was found to be 97.3% (CI 85.8–99.9%), and specificity 95.6% (CI 78.1–99.9%). The combined sensitivity for iOS and Android devices was found to be 91.9% (CI 78.7–97.2%) and specificity was 91.4% (CI 77.6–97.0%). Figure S4. Percent of tests that were observed to have each of the 4 mHAT errors: blood failing to clear from the nitrocellulose membrane (blood clearance), missing control line, physical damage to the test or casing (test defect), or interfering environmental defects. [file 12936_2021_3772_MOESM1_ESM.docx]

**Additional file**

**Additional Methods**

*mHAT Application Software Architecture*

mHAT has a front-end written in HTML (Hypertext Markup Language), CSS (Cascading Style Sheets), and JS (JavaScript). We developed an application programming interface (API) in PHP (hypertext preprocessor) that handles data input/output and server-side processing. This includes user-authentication, transmission of data to and from REDCap, and execution of our computer vision software for RDT analysis. The application uses REDCap as a secure storage database for results and user account information but could readily be ported to another backend database (i.e., MySQL, MongoDB). Our computer vision algorithm (described in detail below) is developed in Python and relies heavily on OpenCV, an open-source computer vision library.

*Computer Vision Algorithm*

Our computer vision software is developed in Python and relies heavily on OpenCV, an open-source computer vision library (Additional Figure 1). Using the web-app, users upload a photograph of the diagnostic test that they would like to analyze. The first step of the algorithm is to identify the test within the picture and to evaluate several metrics (blurriness, brightness, etc.) which can be used to reject the photograph and force the user to take another picture. The algorithm then corrects for uneven illumination (1), and then performs thresholding to facilitate object detection and isolate the RDT within the photograph (2-4). We use the ORB (Oriented FAST and Rotated BRIEF) algorithm to calculate image features and descriptors in the submitted image, which are then matched to a reference image of the particular type of diagnostic test being used (5-6). SURF (Speed up Robust Feature) and SIFT (Scale Invariant Feature Transform) are popular alternative image matching algorithms to ORB. However, these alternatives require a licensing fee which is non-trivial when considering global health applications. After the identification of matching descriptors using Hamming distance as a measure of similarity between query image descriptors and reference image descriptors, we calculate a homography matrix between this set of features for the reference and the query image. From this homography matrix, we can derive several metrics important for analysis and rejection, including translation, rotation, scale, and shear between the query and the reference image. Most critically, we calculate the determinant of the homography matrix: if the matrix is near singular (the determinant is very small), the features of the query image and the reference image are in poor agreement; in this instance, the photograph submitted by the user is rejected and the app requests that the user take another photograph. If the photograph passes this checkpoint, we perform perspective transformation to map the query image onto the dimensions of the reference image (6,7).

Now that the test has been identified in the photograph, and the photograph has been mapped using perspective transformation into a specific orientation with known dimensions, we can perform quantitative image digital image analysis. Using the lamination corrected, perspective transformed image, we perform linescans of hue, saturation, value, and grayscale (8,9). These linsecsans have a width greater than 1 pixel, which averages across the perpendicular direction to avoid image noise or physical contamination of the RDT that could negatively affect signals processing. If the test is valid, the linescan results in an identifiable peak for the control line. The peak of the test line is variable and depends on the concentration of the test’s target antigen in the sample. We use signals processing to locate and identify the locations of these peaks, and our algorithms are tolerant of moderate variations in positioning from test to test and batch to batch of RDTs. Once the control line and test line peaks are identified, we perform numerical integration to calculate a total peak “signal”. As a metric to determine a positive or negative diagnosis of the RDT, we evaluated the integrated test line signal on its own, or the ratio of the test line signal to the control line signal. The threshold of these two metrics was varied in order to generate receiver operating characteristic (ROC) curves and find the optimum values.

For any new test brand, computer vision and image processing features must be experimentally optimized. This is a result of the different shapes of cassettes, the colors of the test and control line signals, markings and brandings on the test cassettes, different spatial differences during manufacturing of each test. However, this process is relatively straightforward, and with minimal effort, can enable the use of the software to analyze a variety of tests. We have performed this optimization for several malaria tests (including multiplexed tests with multiple test lines), multiple HIV test kits, schistosomiasis test kits, pregnancy and ovulation test kits. Since the algorithm utilizes feature recognition, it has the potential to automatically detect the type of test from the photograph, without additional user input. This would have obvious utility for organizations that heavily rely on point-of-care testing.

*mHAT Optimization for Field Performance*

A controlled laboratory environment is an idealized setting for a mobile application that uses computer vision to analyze global health rapid diagnostic tests. To mitigate potential errors in photography resulting from an uncontrolled field setting, we incorporated: 1) an automated checkpoint where the algorithm attempts to automatically determine if a photograph is satisfactory for processing, and 2) a manual checkpoint where a user can determine if the photograph should be retaken. In addition, we anticipated that adjustments to the image-processing algorithms may improve field performance (Additional Figure 2).

**Additional Figures**


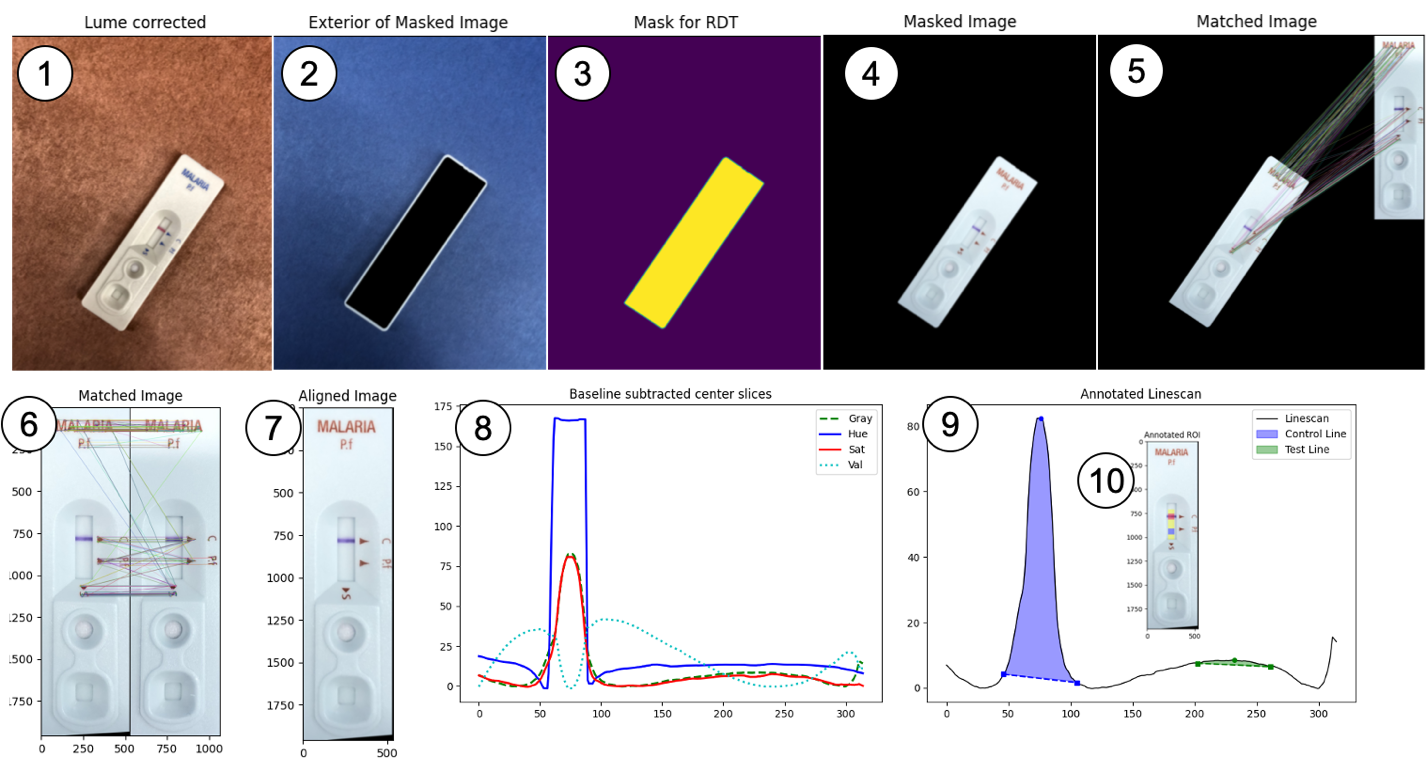


**Additional Figure 1.** The image analysis steps used in the mHAT application.


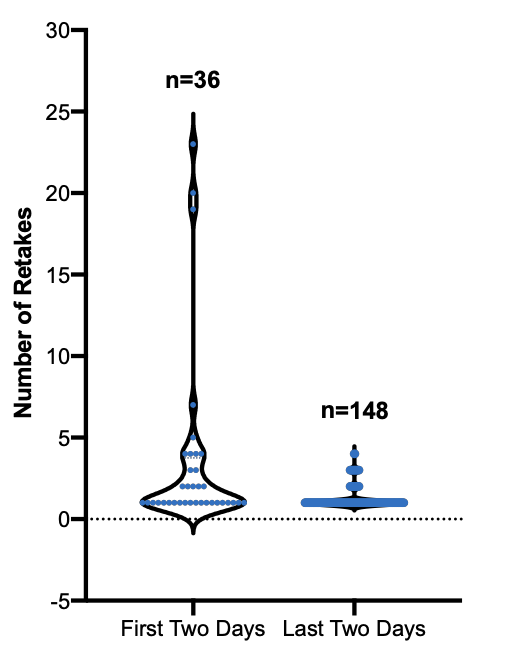


**Additional Figure 2.** Number of photo retakes needed to achieve an mHAT accepted image on any device during the first two days of the field study, and the last two days of the field study. The mean number of retakes needed in the first two days was 3.500 (5.46), compared to 1.284 (0.670) on the final two days of the field trial.

**Additional Figure 3.** Receiver operator characteristic (ROC) curves for the mHAT application, when compared to visual interpretation of RDTs by an experienced healthcare worker, using test line signal as the reporting metric. For iOS devices, sensitivity using test line signal was found to be 91.9% (CI 78.1%-98.3%) and specificity was found to be 91.4% (CI 76.9%-98.2%). Using an Android device, sensitivity with test line signal was found to be 97.3% (CI 85.8%-99.9%), and specificity 95.6% (CI 78.1%-99.9%). The combined sensitivity for iOS and Android devices was found to be 91.9% (CI 78.7-97.2%) and specificity was 91.4% (CI 77.6-97.0%).

**Additional Figure 4.** Percent of tests that were observed to have each of the 4 mHAT errors: blood failing to clear from the nitrocellulose membrane (blood clearance), missing control line, physical damage to the test or casing (test defect), or interfering environmental defects.
